# In Vitro Study on the Effect of a New Bioactive Desensitizer on Dentin Tubule Sealing and Bonding

**DOI:** 10.3390/jfb11020038

**Published:** 2020-06-02

**Authors:** Minh N. Luong, Laurie Huang, Daniel C. N. Chan, Alireza Sadr

**Affiliations:** Department of Restorative Dentistry, University of Washington, Seattle, WA 98195, USA; mndluong@uw.edu (M.N.L.); laurie.whuang@gmail.com (L.H.); dcnchan@uw.edu (D.C.N.C.)

**Keywords:** bioactive desensitizer, hypersensitivity, SEM, nanoparticle, nanohydroxyapatite, microshear bond strength, phosphoric acid, self-etch

## Abstract

Bioactive mineral-based dentin desensitizers that can quickly and effectively seal dentinal tubules and promote dentin mineralization are desired. This in vitro study evaluated a novel nanohydroxyapatite-based desensitizer, Predicta (PBD, Parkell), and its effect on bond strength of dental adhesives. Human dentin discs (2-mm thick) were subjected to 0.5 M EDTA to remove the smear layer and expose tubules, treated with PBD, and processed for surface and cross-sectional SEM examination before and after immersion in simulated body fluid (SBF) for four weeks (ISO 23317-2014). The effects of two dental desensitizers on the microshear bond strength of a universal adhesive and a two-step self-etch system were compared. SEM showed coverage and penetration of nanoparticles in wide tubules on the PBD-treated dentin at the baseline. After four weeks in SBF, untreated dentin showed amorphous mineral deposits while PBD-treated dentin disclosed a highly mineralized structure integrated with dentin. Desensitizers significantly reduced microshear bond strength test (MSBS) of adhesives by 15–20% on average, depending on the bonding protocol. In conclusion, PBD demonstrated effective immediate tubules sealing capability and promoted mineral crystal growth over dentin and into the tubules during SBF-storage. For bonding to desensitizer-treated dentin, a two-step self-etching adhesive or universal bond with phosphoric acid pretreatment are recommended.

## 1. Introduction

Dentin hypersensitivity (DH) has become a more frequently encountered issue in diagnostic and therapeutic clinical situations. DH is identified by a typical short sharp pain on the exposed dentin because of the thermal, evaporative, tactile, osmotic, or chemical stimuli. The common clinical manifestations of DH include exposed dentin from abrasion, erosion or exposed root surface. Although the mechanism of pain transmission and sensitivity in dentin is still under speculation, the most accepted theory is the hydrodynamic mechanism of sensitivity. It is postulated that the sudden flow of tubular fluid within the tubules in the presence of irritating stimulus results in the activation of sensory nerve ending thereby inducing pain or sensitivity [1,2]. The density and size of patent dentinal tubules are critical factors to the severity of DH in patients. 

A standard treatment for DH has not yet been established [3]. Two main methods utilized for the treatment of DH are occlusion of the dentin tubules and interference with the sensitivity of the mechanoreceptor. A plethora of products have been developed with the aim to reduce the discomfort from DH. Dentin desensitizers have been introduced for DH treatment mainly through tubular occlusion [4,5,6,7]. Calcium phosphate mineral-based desensitizers have attracted considerable interest in recent years thanks to their biocompatible properties yielding an effective dentinal tubule occlusion and reduction in dentin permeability. They also possess the possibility of mineral crystal growth in the oral environment [8,9,10]. The composition of calcium and phosphate in a material may exhibit the ability to form hydroxyapatite (HA; Ca_10_(PO_4_)_6_(OH)_2_) as a final product. HA and its variations are the main mineral component in human tooth and their biocompatibility have rendered them as clinically practical choices [11].

Dental adhesives have been utilized in the treatment of restoring teeth in combination with DH. The component in the bonding agent provides the simultaneous conditioning and priming for the underlying dentin, resulting in superior bond strength and reduced DH [12,13]. However, the sealing ability of adhesives on the dentin surface could be affected by an incomplete micro-emulsion polymerization of the hydrophilic primer and hydrophobic bonding [14]. Therefore, in order to increase the effectiveness of treatment, it would be necessary to desensitize dentin exhibiting DH prior to the placement of a restoration. It was reported that one-step self-etching adhesives and dentin desensitizers could significantly relieve DH immediately and over a month after treatment [15]. The usage of dentin desensitizer in the combination with the bonding agent can alleviate the postoperative sensitivity with the composite restoration [16]. Nevertheless, its effect on bonding performance using different bonding systems remains to be evaluated, as the desensitized treated dentin may be unfavorable for bonding [17,18]. The objective of this in vitro study was to evaluate the morphological characteristic of the new nanohydroxyapatite-based desensitizer with the proprietary composition of calcium, sodium, phosphate, and silica when applied on the tooth surface and the effect of this material on the bond strength. The null hypotheses were that the novel desensitizer could modify the morphological characteristic of the dentin of the extracted teeth and did not interfere with the bonding performance of the tested adhesives.

## 2. Materials and Methods

The extracted human teeth were used in this study in accordance with the guideline of the Ethics Committee of the University of Washington Human Subject Division and the Declaration of Helsinki, which allow the use of extracted deidentified human teeth. Forty molars were collected during treatment plan and patient care and stored at 4 °C in water with 0.02% thymol. 

The occlusal cusps were removed by cutting perpendicularly to the long axis of the tooth 2–3 mm from the tip of the cusp to expose superficial midcoronal dentin using a low-speed water-cooled diamond saw (Isomet; Buehler, Lake Bluff, IL, USA). The roots were then sectioned at the cement-enamel junction to obtain 2-mm-thick dentin slices. The occlusal side of each disc was polished under running water by 600-grit paper (3M, St. Paul, MN, USA) for 30 s in circular motion to create a standardized smear layer. The surfaces were observed under the digital stereomicroscope (MU1000, AmScope, Irvine, CA, USA) to eliminate the cracks, residual enamel, coarseness and pollutants.

The composition and application of desensitizers and adhesives according to the manufacturer’s instruction were listed in Table 1. 

### 2.1. Morphological Observation

**Figure 1:**
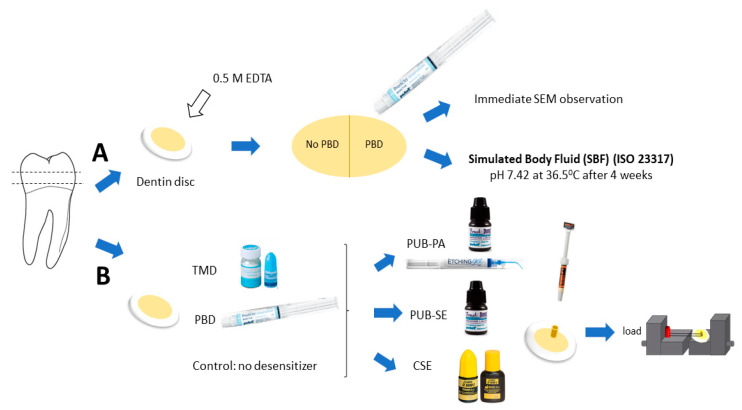
Methodology: Dentin discs were produced from human molar for morphological observation (**A**) and microshear bond strength test (**B**). For (**A**): Ten dentin discs were pretreated with EDTA (pH 7.4) to remove the smear layer and open dentinal tubules before rinsing. Half of the disc surfaces were subjected to Predicta Bioactive Desensitizer (PBD) and the other half served as control (no PBD). Five discs were immediately processed for SEM examination while the other five were stored for four weeks in simulated body fluid (SBF) for bioactivity test (specified by the ISO 23317:2014). For (**B**): Thirty dentin discs were allocated into three groups (n = 10): Teethmate Desensitizer (TMD) treatment, PBD treatment and no desensitizer as control. The universal adhesive (PUB) in self-etch (SE) or with phosphoric acid etching (PA)) and two-step self-etch adhesive Clearfil SE Bond 2 (CSE) were used. Small cylinders of the hybrid composite were bonded on the dentin surface and the wire loop bond test was performed at a crosshead speed of 1 mm/min.

Ten dentin discs were pretreated with 0.5 M EDTA (pH 7.4) for 2 min to completely remove the smear layer and widely open the dentinal tubules before applying the deionized water. Half of the disc surfaces were subjected to the Predicta Bioactive Desensitizer (PBD, Parkell, Edgewood, NY, USA) whereas the other half served as control (no PBD). Five discs were immediately processed for SEM examination while the other five were stored for four weeks in simulated body fluid (SBF) for bioactivity test (specified by the ISO 23317:2014) (Figure 1A).

### 2.2. Observation after Application of PBD

The specimens were dried in a desiccator without heating for 24 h. The discs were then superficially scored using a diamond bur and delicately fractured by finger pressure revealing the cross-section of dentinal tubules without creating an additional smear layer. The boundary between applied and non-applied dentin surface was targeted for visualization under SEM (JEOL JSM-6010LA, Tokyo, Japan). After gold-sputter coating, the micromorphology was examined at magnifications of 1000×, 4000×, 5000×, and 10,000×. Furthermore, in order to anticipate the bioactivity of PBD-treated dentin in the oral environment, the dentin discs were further placed in dust-free SBF solution incubated at 37 °C for four weeks in a transparent bottle. 

### 2.3. Microshear Bond Strength Test

Thirty dentin discs were randomly distributed into three groups (n = 10): Teethmate Desensitizer (TMD, Kuraray Noritake Dental Inc, Tokyo, Japan) treatment, Predicta Bioactive Desensitizer (PBD) treatment and no treatment (control). The universal adhesive PBOND (PUB, Parkell, Edgewood, NY, USA) in self-etch (SE) or with phosphoric acid etching (PA, Parkell Etching Gel, Parkell, Edgewood, NY, USA) and two-step self-etch adhesive Clearfil SE Bond 2 (CSE, Kuraray Noritake Dental) were used in the experiment of microshear bond strength test (MSBS). Figure 1 represented the method of the study.

Prior to the irradiation, three pieces of Tygon tubes (Saint-Gobain Performance Plastic, Nagano, Japan) with an internal diameter of 0.8 mm and 1.0 mm in height were fixed on the dentin surface of each slice. The adhesive was light-cured for 10 s at 600 mW/cm^2^ (Yoshida Light Curing Unit, Japan). The hybrid composite Z100 (A2 Shade, 3M ESPE, St. Paul, MN, USA) was carefully placed inside the tubing lumens before irradiation for 40 s. After 1 h-storage at room temperature (25 °C), the tygon tubes were removed using a sharp blade to obtain the cylinders of resin with the size of 0.8 mm in diameter and 1.0 mm in height bonded to the dentin surface. The specimens were then stored in distilled water in an incubator at 37 °C.

After storage, an MSBS test was conducted. The dentin slice was attached to the testing apparatus (Mecmesin, Compact Force Gauge, CFG+, UK) with a thin layer of cyanoacrylate glue (Model Repair II Blue, Dentsply-Sankin, Tochigi, Japan) and tested in a microshear tester (Bisco, Inc., Schaumburg, IL, USA). A 0.2 mm-diameter steel wire was looped at the bottom of the resin cylinder, in contact with the lower half-circle of the cylinder and the dentin surface. The wire loop was maintained according to the shear stress orientation at the bonding interface. The force was applied at a crosshead speed of 1 mm/min until failure then the MSBS data were recorded (Figure 1B).

### 2.4. Evaluation of Failure Mode

After the MSBS test, the mode of failure was determined for each specimen under the digital stereomicroscope. The fractured surfaces were classified as followed: adhesive failures between resin and dentin, mixed failures that were partially adhesive and partially cohesive, and cohesive failures that occurred entirely within the bonding resin or entirely in dentin.

### 2.5. Statistical Analysis

The distribution of data in each group was normal (Kolmogorov–Smirnov *p* > 0.05), parametric tests were performed. The MSBS values were statistically analyzed using two-way ANOVA with application mode and desensitizer treatment as factors. The statistical procedures were analyzed at 0.05 significance level using IBM SPSS Statistics 23 Software. 

## 3. Results

**Figure 2:**
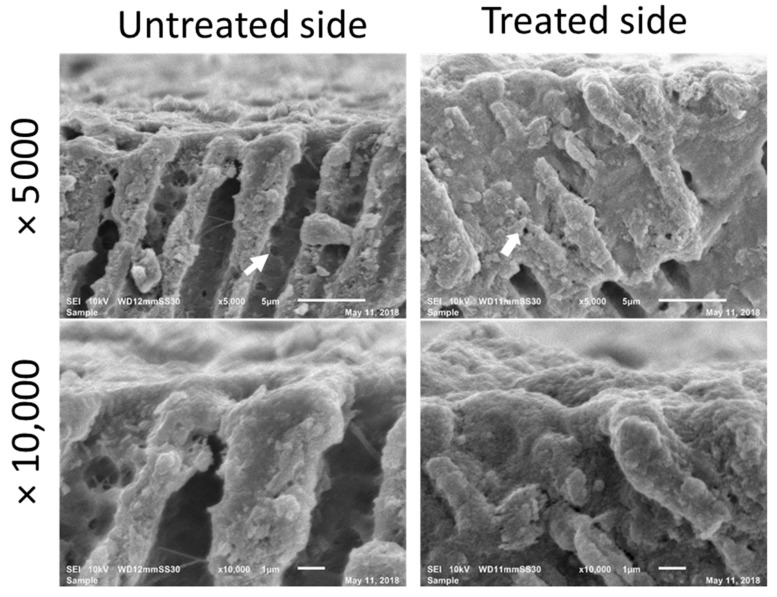
Cross-sectional SEM images immediately after application of PBD. The patency of dentinal tubules and open lateral branch orifice (arrow) have been well sealed on the treated side.

Immediately after the application of PBD, SEM micrographs showed good coverage by PBD of the widely open dentinal tubule in EDTA-demineralized dentin as clearly observed in the comparison of the untreated and treated side (Figure 2). The cross-sectional images showed that the nanoparticles form PBD have penetrated into the dentinal tubules and further sealed the nanospaces exposed through the collagen network and lateral branches in the tubule walls of demineralized dentin. Intratubular dentin seemed to be densely penetrated on the treated side. The depth of penetration was estimated to be 5–10 µm from the dentin surface. The thickness of the formed layer as a result of the PBD application was approximately 1 µm.

**Figure 3:**
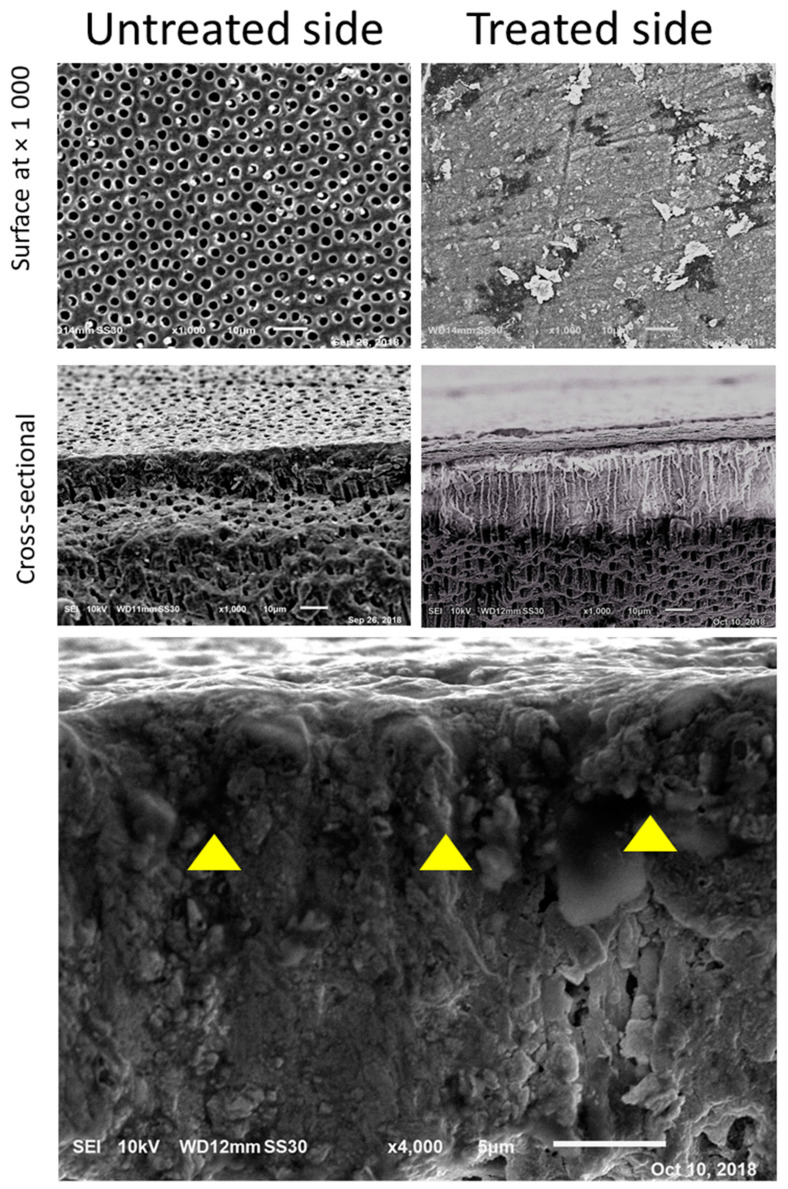
SEM images four weeks after application of PBD and immersion in SBF. PBD-treated dentin demonstrates a solid layer of mineral formation while no coverage is distinguished in the untreated dentin away from the PBD-treated dentin.

After four weeks of SBF immersion, SEM images of dentin treated with EDTA and PBD disclosed a highly dense and homogenous structure of dentin measuring at least 20 µm in thickness. In this structure, a surface layer was not easily distinguished from the bulk of subsurface dentin, indicating integrated mineralization of the surface zone through both mineral deposition on the surface and into the demineralized dentin, forming a highly mineralized unified structure whereas there was no such unification on untreated sides (Figure 3). 

**Figure 4:**
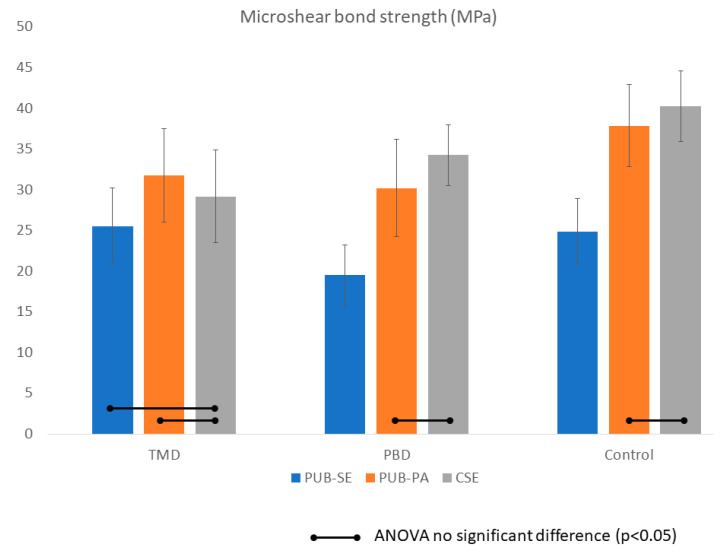
Bond strength of the experiment group in MPa. Bars indicate no significant difference (*p* < 0.05). Two-way ANOVA indicated that both the use of desensitizers and application mode were significant factors in MSBS and their interaction was significant (*p* < 0.05). Predicta (PBD) and Teethmate (TMD) significantly reduced bond strength to dentin by 15%–20% on average; however, the effect depended on the adhesives. For Universal Adhesive (PUB), the groups using phosphoric acid etching (PUB-PA) consistently showed higher bond strength compared to the ones using self-etch (PUB). Bonding to PBD and TMD with either PUB-PA and Clearfil SE 2 (CSE) were comparable (*p* > 0.05).

The results of MSBS of each experimental group (mean and SD) were listed in Table 2 and Figure 4. Two-way ANOVA indicated that both the use of desensitizers and application mode were significant factors in MSBS and their interaction was significant (*p* < 0.05). Generally, both PBD and TMD significantly reduced bond strength to dentin by 15–20% on average; however, the effect was adhesive dependent. For PUB, the groups using phosphoric acid etching (PUB-PA) consistently showed higher bond strength compared to the ones using self-etch (PUB). Bonding to PBD and TMD with either PUB-PA and CSE were comparable (*p* > 0.05). 

## 4. Discussion

Desensitizing agents occlude the dentinal tubules at the tubular orifice or within the dentinal tubules to prevent the fluid flow and therefore decrease the pain sensation by counteracting the hydrodynamic mechanism of DH. The effectiveness of various desensitizing agent to mitigate the DH has been reported in previous studies. The clinical efficacy has been reported in the application on vital abutment teeth prepared to receive full coverage or porcelain fused to metal restoration [19,20]. The pre-impression sealing of the dentin was recommended for tooth preparation on vital teeth to reduce the pressure transmitted to the pulp chamber during the crown cementation [21]. 

In this study, immediately after the application of PBD, the untreated dentin showed patent dentinal tubules. At the area adjacent to the PBD-treated dentin, random mineral deposits were observed on the untreated dentin. It is assumed that these deposits have formed as a result of free PBD nanoparticles adjacent to the PBD-treated dentin (Figure 3).

For evaluation of the mineral forming ability of PBD-treated dentin, SBF immersion proceeded for four weeks. The SBF was used to simulate the clinical situation where dentin interacts with body fluids, such as saliva from the oral environment and dentinal fluid and plasma from the pulpal side. The SBF is an acellular and protein-free simulated fluid with ion concentration nearly equal to those of human blood plasma. SBF was preferred over artificial saliva formulations for the bioactivity experiment in this study since the ISO test has standardized the SBF for bioactivity test and apatite formation ability, while there is not a comparable standard established for artificial saliva formulations. Due to the similarity between bone and dentin in terms of mineral composition and structure, the bioactivity test for bone implant devices would be applicable to a desensitizer material applied to dentin. The bioactive material is expected to form a layer of apatite on their surface [22]. In this study, PBD demonstrated bioactivity as defined by the ISO 23317-2014 document. Considering the composition of SBF, the forming mineral in the ISO experiment is expected to be apatite. 

The SEM micrographs confirmed that the dentin regions exposed for the experiment were rich in open dentin tubules, and tubular sealing was achieved immediately by PBD. The mineral growth matured through the SBF-storage period. Fine precipitates are observed inside the tubules close to the surface whereas the group without PBD, there were patent tubules. The principal mineral content in PBD (calcium and phosphate, as disclosed by the manufacturer) could stimulate the mineralization of the dentine tubules. During immersion in SBF, different processes occur resulting in structural and chemical change at the surface of dentin: leaching, degradation, and precipitation. Calcium ions dissolve from the bioactive substance into the body fluid. The nucleation of hydroxyapatite is possible because the surrounding fluid is supersaturated with respect to hydroxyapatite due to the dissolution of the calcium ions. The process of formation and growth of the hydroxyapatite layer continues by the reactions of calcium, phosphate and hydroxide ions [23]. In the oral cavity, the supersaturated calcium phosphate salts contained in the saliva could precipitate to form a less soluble compound HA continuously [24].

The study compared the bond strength of adhesives to dentin treated with PBD and TMD. The main components of TMD are TTCP and DCPA, which are eventually converted to hydroxyapatite in an aqueous environment. This conversion is predicated upon the dissolution-precipitation reaction mechanism [25]. TTCP and DCPA dissolve and supply Ca^2+^ and PO_4_^3−^. The supersaturation of the apatite-contained solution prompted the precipitation of HA crystals. TMD has been investigated previously in vitro with a superior tubule occlusion rate compared to silver diamine fluoride and resin-containing oxalate [6,8,26]. SEM images of TMD showed all tubules occluded with crystalline precipitates, with similar precipitation on the intertubular surface [7]. The application of TMD occluded dentinal tubules and reduced dentin permeability by 92% regardless of the exposed collagen network [26]. A comparable tubule occlusion was found in the novel PBD, therefore its sealing ability for treatment DH is promising. 

As far as bonding performance is concerned, dentin desensitizers significantly decreased the bond strength of bonding agents. These findings are in alignment with the results of previous work [9,16,18]. The application of desensitizers before the bonding system significantly reduced shear bond strengths to sound dentin. In the three-step bonding system, the protocol included acid etching, rinsing, and drying of dentin before applying the primer. Self-etching primers succeeded in eliminating the dentin-conditioning steps before priming. Nevertheless, the majority of self-etching primers have a milder acidity. Therefore, the lower bond strengths to sound dentin might be accounted for the desensitizer deposition blocking the dentin tubule orifices and intertubular diffusion channels. The nanoparticles layer of the desensitizer dispersed on the dentin could hinder the adhesives to interact with the demineralized substrate, resulting in reducing the bond strength [16]. 

The literature has reported that the dentin treated with mineral-based desensitizers did not show a reduction in the bond strength when treated with a two-step self-etching adhesive material [27]. The reason for this phenomenon was explained by the interaction created between the desensitizer, the smear layer, and dentin. This was in contrast with the result in this present study where bond strength in PUB-SE mode after application of PBD and TMD was reduced compared to the control group. 

The functional monomer in self-etch adhesives could contribute to the superficial micromechanical interlocking through hybridization of dentin substrate. The exposed HA crystal that remains around collagen is expected to be beneficial, as a substrate for chemical bonding. A mineral-rich substrate allow a more substantial chemical interaction with the functional monomer and protect the collagen against hydrolysis thus prevent from premature degradation [28,29].

It was of interest to compare the bond strength characteristics of two products PBD and TMD in a combination of adhesives. The result indicated that bonding to PBD with PUB-PA and CSE is comparable to that to TMD. The bonding performance of the PBD-treated specimen was corroborated with SEM images obtained by the application of PUB on dentin. PUB with acid etching exhibited resin tag formation, which could explain higher values in groups bonded in PA mode. Nonetheless, it was reported that the sequence of application of etching and desensitizer affected the bonding performance [16,30]. When dentin was acid-etched before the application of oxalate desensitizer [16] or Novamin [30], the bonding performance was not compromised. However, in this application sequence, the formation of resin tags might be affected by tubule occlusion with calcium-containing desensitizer pastes [30]. The clinical decision of whether to etch dentin or not after desensitizer application should be made considering the potential increase in permeability of dentin following etching. The interaction of PBD and phosphoric acid etched dentin, as application of the acid prior to treatment with PBD and the universal bond, warrants further investigation.

Successful bonding to dentin may further reduce the effects of hydraulic pressure for the treatment of the post-operative DH [31,32]. The use of the universal bonding system with acid phosphoric etching or the two-step self-etching is recommended following the usage of mineral-based desensitizer to achieve a high bond strength. 

The study highlights the biomimetic remineralization shown by desensitizing agent PBD. It could be of interest to compare investigate the characteristics of PBD in vivo. The present laboratory test could be to some extent a predictive tool for the clinical efficacy of desensitizing materials, while the effect of adhesive systems on the survival rate of restorations following desensitizing treatment should be further studied.

## 5. Conclusions

In this in vitro study, the newly developed dentin desensitizer demonstrated effective dentinal tubules sealing capability and promoted mineral crystal growth over dentin and into the tubules during simulated body fluid storage. For bonding to the desensitizer-treated dentin, dentin treatment by two-step self-etching system or universal bond with phosphoric acid is recommended.

## Figures and Tables

**Table 1:** Materials used in the study.

Materials	Composition	Application
Teethmate Desensitizer (TMD) (Kuraray Noritake Dental Inc, Tokyo, Japan)	Powder: tetracalcium phosphate (TTCP) and dicalcium phosphate anhydrous (DCPA) Liquid: water	Mix powder and water within 30 s, apply slurry with microapplicator 15 s, rub 60 s rinse with water spray.
Predicta Bioactive Desensitizer gel (PBD, Parkell, Edgewood, NY, USA)	Calcium, phosphate, nanohydroxyapatite	Rinse surface with warm water, dry with absorbent cotton. Paint a coat and rub gently the liquid in 10–20 s using an applicator. Wipe the product off using a cotton pledget before air blowing.
Parkell Etching (Parkell, Edgewood, NY, USA)	32% PA Gel (H_3_PO_4_)	10–15 s etch then rinse.
Parkell Universal Adhesive PBOND (Parkell, Edgewood, NY, USA)	Acetone, Ethyl Alcohol, 10-MDP, 2-Hydroxylethyl methacrylate, 2-Propenoic acid	Mix well, dispense 1–3 drops. Rub the liquid using an applicator, keep moist for 20 s. Blow gently for 10 s and light cure for 10 s.
Clearfil SE 2 (Kuraray Noritake Dental, Japan)	Primer: MDP, HEMA, camphorquinone, hydrophilic dimethacrylate, N,N-diethanol p-toludine, water. Bond: MDP, Bis-GMA, HEMA, camphorquinone, hydrophobic dimethacrylate, N,N-diethanol p-toludine, silanated colloidal silica.	With the applicator, prime for 20 s. Apply bond and light cure for 10 s
Z100 Restorative (3M ESPE, St. Paul, MN, USA)	Bis-GMA, TEDGMA, Zirconium/Silica filler	Build the resin cylinder then light cure for 40 s.

**Table 2:** Mean microshear bond strength of the experimental groups (MPa).

Mean ± SD.	TMD	PBD	Control
PUB-SE	25.5 ± 4.7	19.5 ± 3.7	24.9 ± 4.0
PUB-PA	31.8 ± 5.8	30.2 ± 5.9	37.9 ± 5.0
CSE	29.2 ± 5.7	34.2 ± 3.7	40.3 ± 4.3
